# Network biology analysis of P23H rhodopsin interactome identifies protein and mRNA quality control mechanisms

**DOI:** 10.1038/s41598-022-22316-8

**Published:** 2022-10-18

**Authors:** Kyle Kim, Lance A. Safarta, Wei-Chieh J. Chiang, Judith A. Coppinger, Eun-Jin Lee, Jonathan H. Lin

**Affiliations:** 1 Stanford University School of Medicine, 300 Pasteur Dr. L235, Stanford, CA 94305 USA; 2 VA Palo Alto, Palo Alto, CA 94304 USA; 3 University of Southern California, Los Angeles, CA USA; 4 Developmental Neurobiology Unit, Okinawa Institute of Science and Technology Graduate University, Okinawa, Japan; 5 School of Pharmacy and Biomolecular Sciences, Royal College of Surgeons in Ireland (RCSI), Dublin, Ireland

**Keywords:** Protein folding, Eye diseases

## Abstract

Rhodopsin is essential for phototransduction, and many rhodopsin mutations cause heritable retinal degenerations. The P23H rhodopsin variant generates a misfolded rhodopsin protein that photoreceptors quickly target for degradation by mechanisms that are incompletely understood. To gain insight into how P23H rhodopsin is removed from rods, we used mass spectrometry to identify protein interaction partners of P23H rhodopsin immunopurified from *Rho*^*P23H/P23H*^ mice and compared them with protein interaction partners of wild-type rhodopsin from *Rho*^+*/*+^ mice. We identified 286 proteins associated with P23H rhodopsin and 276 proteins associated with wild-type rhodopsin. 113 proteins were shared between wild-type and mutant rhodopsin protein interactomes. In the P23H rhodopsin protein interactome, we saw loss of phototransduction, retinal cycle, and rhodopsin protein trafficking proteins but gain of ubiquitin-related proteins when compared with the wild-type rhodopsin protein interactome. In the P23H rhodopsin protein interactome, we saw enrichment of gene ontology terms related to ER-associated protein degradation, ER stress, and translation. Protein–protein interaction network analysis revealed that translational and ribosomal quality control proteins were significant regulators in the P23H rhodopsin protein interactome. The protein partners identified in our study may provide new insights into how photoreceptors recognize and clear mutant rhodopsin, offering possible novel targets involved in retinal degeneration pathogenesis.

## Introduction

Rhodopsin is a G-protein-coupled receptor class protein that is essential for vision and is expressed in rod photoreceptor cells^1^. *Rhodopsin* mRNA is translated as a 348-amino acid polypeptide at the endoplasmic reticulum (ER) organelle^1^. Newly synthesized rhodopsin protein loops into a 7-transmembrane receptor conformation, and once correctly folded, rhodopsin exits the ER and traffics from the rod inner segment to the rod cell cilium/basal body, where it is densely packaged into membranous discs^2,3^. Newly formed discs are then delivered to the rod outer segment^1^. Rhodopsin in the outer segment turns over when retinal pigment epithelia phagocytizes the distal tips of rod outer segments and degrades the engulfed disc contents containing aged rhodopsin^4,5^. Rhodopsin constitutes ~ 90% of all proteins in the outer segment, and rods express vast amounts of rhodopsin throughout life to meet this large demand^1,6–8^.

Over 150 mutations in rhodopsin have been identified that cause rod photoreceptor dysfunction and cell death and lead to clinical symptoms of retinitis pigmentosa and other blinding diseases (RetNet, Retinal Information Network, https://sph.uth.edu/retnet/disease.htm). The first retinitis pigmentosa (RP) mutation, found in 1990, introduced a missense mutation that converted proline to histidine at amino acid 23 (P23H) of rhodopsin^5^. Transgenic drosophila^9^, Xenopus^10^, rat^11^, pig^12^, and mouse^13^ models of RP have been generated through over-expression of P23H rhodopsin. More recently, P23H RP “knock-in” mouse models have been generated through introduction of a P23H conversion directly in the native mouse rhodopsin locus (*Rho*^*P23H/*+^)^14^, as well as fluorophore-tagged P23H rhodopsin (P23H-GFP^15^, or P23H-RFP^16^) models. These animal models provide tools to study pathomechanisms underlying rod cell death and to explore strategies to prevent retinal degeneration.

The P23H amino acid conversion results in damaged rhodopsin protein structure and function. P23H rhodopsin cannot couple efficiently with retinal to generate rod visual pigment^17^. P23H rhodopsin forms higher order protein aggregates ^17–20^. P23H rhodopsin is retained in the ER instead of trafficking down the secretory pathway^17^. P23H rhodopsin is heavily ubiquitinylated and degraded^19,21^. Several post-translational protein quality control mechanisms, ER-associated protein degradation (ERAD) and autophagy, are implicated in degradation of P23H rhodopsin. ERAD involves retro-translocation of misfolded membrane or secreted proteins from the ER to the cytosol for ubiquitylation and 26S proteasomal degradation^22^. ERAD is induced as part of the Unfolded Protein Response (UPR) to restore ER homeostasis and cellular health by reducing misfolded protein levels^23^. *Rho*^*P23H/*+^ mice crossed with *ERAI* mice (carrying a GFP reporter of UPR activity^24^) or Ub-GFP mice (carrying a GFP reporter of proteasome activity^25^) showed selective GFP induction in rods expressing P23H rhodopsin^19,26,27^. Genetic and chemical activation of UPR, ERAD, or proteasome regulators promoted P23H rhodopsin degradation in vitro^9,19–21,28–31^ and in vivo^32^. Deletion or inhibition of UPR regulatory genes impaired P23H rhodopsin clearance in *Rho*^*P23H/*+^ mice^33^ and rats^34^. By contrast to ERAD, autophagy degrades proteins by encapsulating targets in double-membrane vesicles that then fuse with the lysosome and release their cargo for breakdown by lysosomal enzymes^35^. Genetic and chemical modulation of autophagy also supports a role for this catabolic mechanism in clearance of P23H rhodopsin^36^. Interestingly, photoreceptor function improved in *Rho*^*P23H/*+^ mice with modulation of UPR^37,38^, proteasome function^32^, or autophagy^36^. These findings support that these protein quality control mechanisms, when properly harnessed, may prevent retinal degeneration in RP patients carrying misfolded rhodopsin.

To advance the therapeutic potential of protein quality control mechanisms to treat RP, it is important to identify the proteins that photoreceptors employ to recognize and target misfolded rhodopsin, but not normal rhodopsin, for degradation by ERAD, autophagy, or other catabolic processes. In this study, we immunopurified native P23H rhodopsin from homozygous P23H knock-in mouse (*Rho*^*P23H/P23H*^) retinas and identified 200+ P23H rhodopsin protein binding partners by mass spectrometry. In parallel, we also immunopurified wild-type rhodopsin from *Rho*^+*/*+^ mouse retinas and identified wild-type rhodopsin interacting proteins. We found significant differences in the P23H and wild-type rhodopsin protein interactomes that correlate with previously identified physiologic differences. Network pathway analysis confirmed that ER stress and ERAD are significant protein quality control processes preferentially associated with P23H rhodopsin and pointed to previously unstudied quality control steps targeting P23H rhodopsin during earlier translational and co-translational steps of biogenesis. These P23H and wild-type rhodopsin proteomic datasets from native retinas provide a resource for additional studies about rhodopsin protein quality control, rod cell biology, and pathomechanisms underlying retinitis pigmentosa.

## Results

### Generation of wild-type and P23H rhodopsin retinal protein interactomes

**Figure 1:**
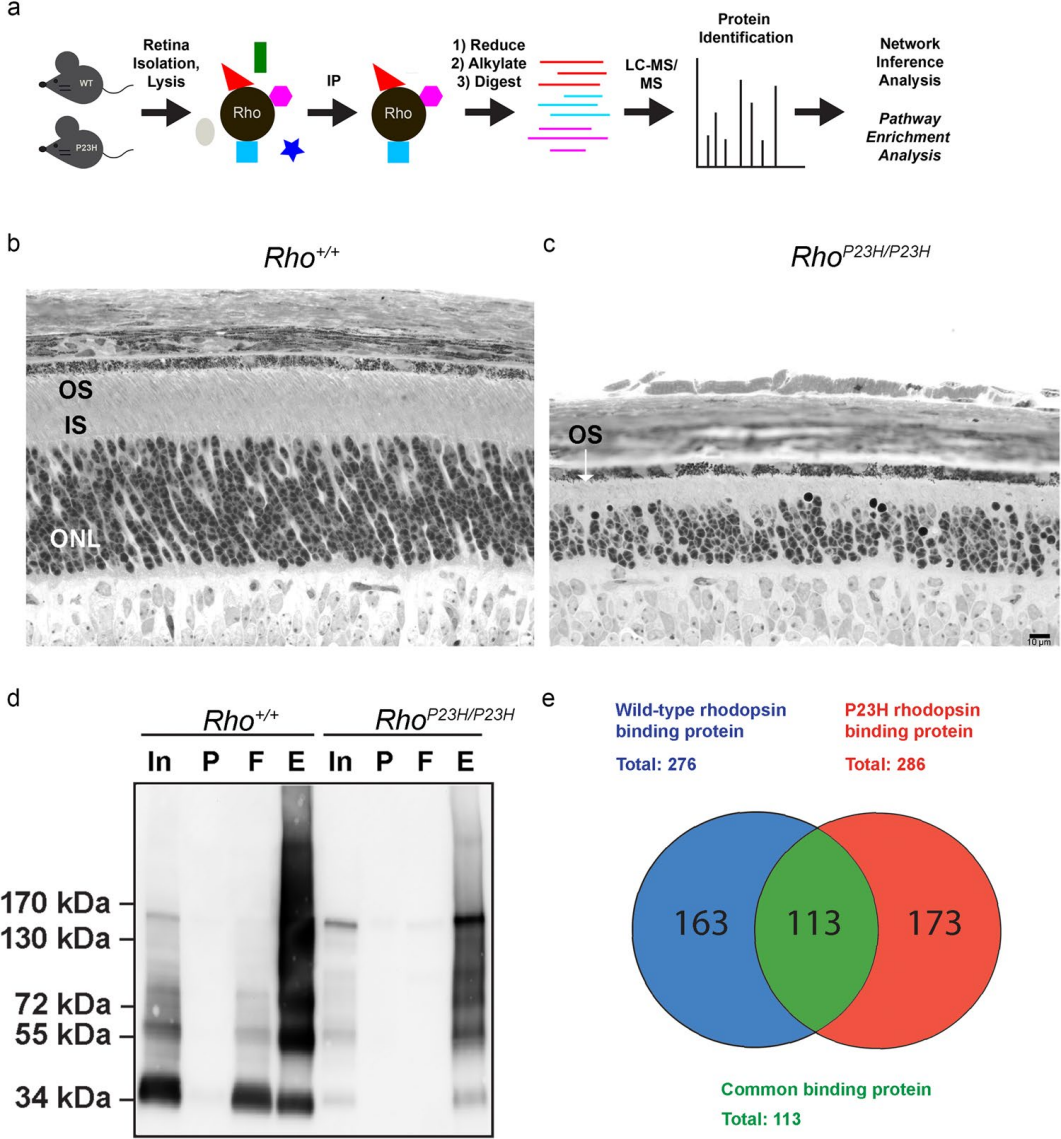
Identification of wild-type and P23H rhodopsin-interacting proteins from *Rho*^+*/*+^ and *Rho*^*P23H/P23H*^ mouse retina. (**a**) Graphical summary of the experimental design. Liquid chromatography-mass spectrometry (LC–MS/MS) was performed on wild-type or P23H rhodopsin immunopurified from *Rho*^+*/*+^ or *Rho*^*P23H/P23H*^ mouse retinas. (**b, c**) Light micrographs of *Rho*^+*/*+^ (**b**) and *Rho*^*P23H/P23H*^ (**c**) mouse retinas at postnatal day 15 (scale represents 10 µm). The white arrow refers to the location of the degenerating outer segment (OS) in *Rho*^*P23H/P23H*^ mice. IS, inner segment. ONL, outer nuclear layer. (**d**) Western blots of *Rho*^+*/*+^and *Rho*^*P23H/P23H*^ P15 mouse retina protein lysate fractions probed for rhodopsin (anti-rhodopsin, 1D4). In, input. P, pellet. F, flow-through. E, eluate. Uncropped blots are shown in Supplementary Information 1. (**e**) Venn diagram of 163 unique proteins found associated to wild-type rhodopsin and 173 unique proteins associated to P23H rhodopsin by LC–MS/MS. 113 proteins were common to both wild-type rhodopsin and P23H rhodopsin.

To identify proteins that interact with wild-type rhodopsin or P23H rhodopsin in photoreceptors, we performed mass spectrometry on rhodopsin immunopurified from mouse retinas (Fig. 1a). We harvested retinas from *Rho*^+*/*+^ mice that express only wild-type rhodopsin protein and from *Rho*^*P23H/P23H*^ mice that express only P23H rhodopsin. *Rho*^*P23H/P23H*^ mice undergo rapid retinal degeneration, and by post-natal day 30, nearly all photoreceptors are lost^14,19^. Therefore, we collected retinas at an earlier post-natal day 15 (P15) when viable photoreceptors are still present in *Rho*^*P23H/P23H*^ mice for our proteomic studies (Fig. 1b,c). We separately pooled and solubilized retinas from either 24 *Rho*^+*/*+^ or 40 *Rho*^*P23H/P23H*^ mice to generate retinal protein lysates (Fig. 1d, Supplementary Information S1). Next, we immunoprecipitated wild-type rhodopsin and P23H rhodopsin from lysates using 1D4 anti-rhodopsin antibody^39,40^. Immunoblotting for rhodopsin confirmed abundant recovery of wild-type and P23H rhodopsin protein in our immunoprecipitation eluates (Fig. 1d, Supplementary Information S1). Next, we performed liquid chromatography tandem mass spectrometry (LC–MS/MS) on triplicate samples of immunopurified wild-type and P23H rhodopsin protein eluates.

As expected, wild-type rhodopsin or P23 rhodopsin was the most frequently found protein in both mass spectrometry experiments (Supplementary Information S2 and S3), and the identified polypeptide fragments covered approximately 1/3 of the wild-type rhodopsin or P23H rhodopsin full-length sequence (Table 1). The 9-amino acid carboxyl termini, specifically recognized by the 1D4 antibody, was identified in both wild-type and P23H rhodopsin mass spectrometry datasets (Table 1). In addition to rhodopsin, we also identified 276 proteins in the wild-type rhodopsin LC–MS/MS and 286 proteins in the P23H rhodopsin LC–MS/MS (Fig. 1e, Supplementary Information S2 and S3). Interestingly, we found significant differences in the wild-type and P23H rhodopsin protein interactomes: 163 proteins were unique to the wild-type rhodopsin protein interactome; 173 proteins were unique to the P23H rhodopsin interactome; and 113 proteins were common between both interactomes (Fig. 1e).

**Table 1:**
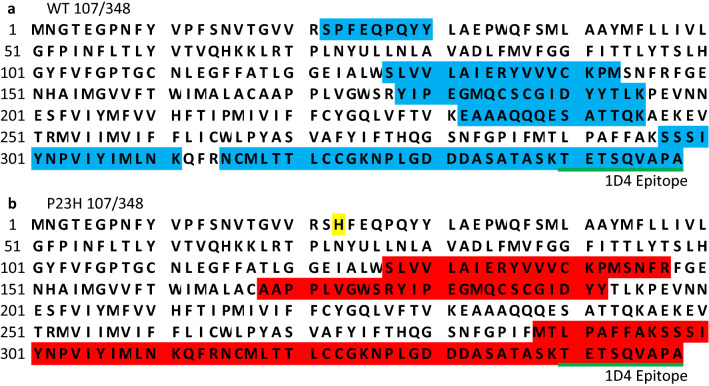
Wild-type and P23H rhodopsin amino acid coverage. a. The wild-type rhodopsin sequence (107/348 amino acids) identified by mass spectrometry is highlighted in blue. b. The P23H rhodopsin sequence (107/348 amino acids) identified by mass spectrometry is highlighted in red. Highlighted in yellow is the P23H mutation. The location of the 1D4 anti-rhodopsin epitope is underlined in green.

To help assess the specificity of our rhodopsin-interacting proteins, we also performed LC–MS/MS on triplicate samples of mouse embryonic fibroblast (MEF) protein lysate eluates immunopurified with 1D4. MEFs do not express rhodopsin, and this was experimentally confirmed by our LC–MS/MS, which showed no rhodopsin protein fragments (Supplementary Information S2 and S3). Furthermore, the vast majority of the proteins identified in our wild-type and P23H rhodopsin interactomes were absent in the MEF LC–MS/MS results (Supplementary Information S2 and S3). Together, these results support that we recovered high quality wild-type and P23H rhodopsin from native whole retinas and identified rhodopsin protein-interacting partners found in mouse retinas.

### P23H rhodopsin interactome is depleted in phototransduction, retinal cycle, and rhodopsin trafficking components but enriched in ubiquitinoylation components

**Figure 2:**
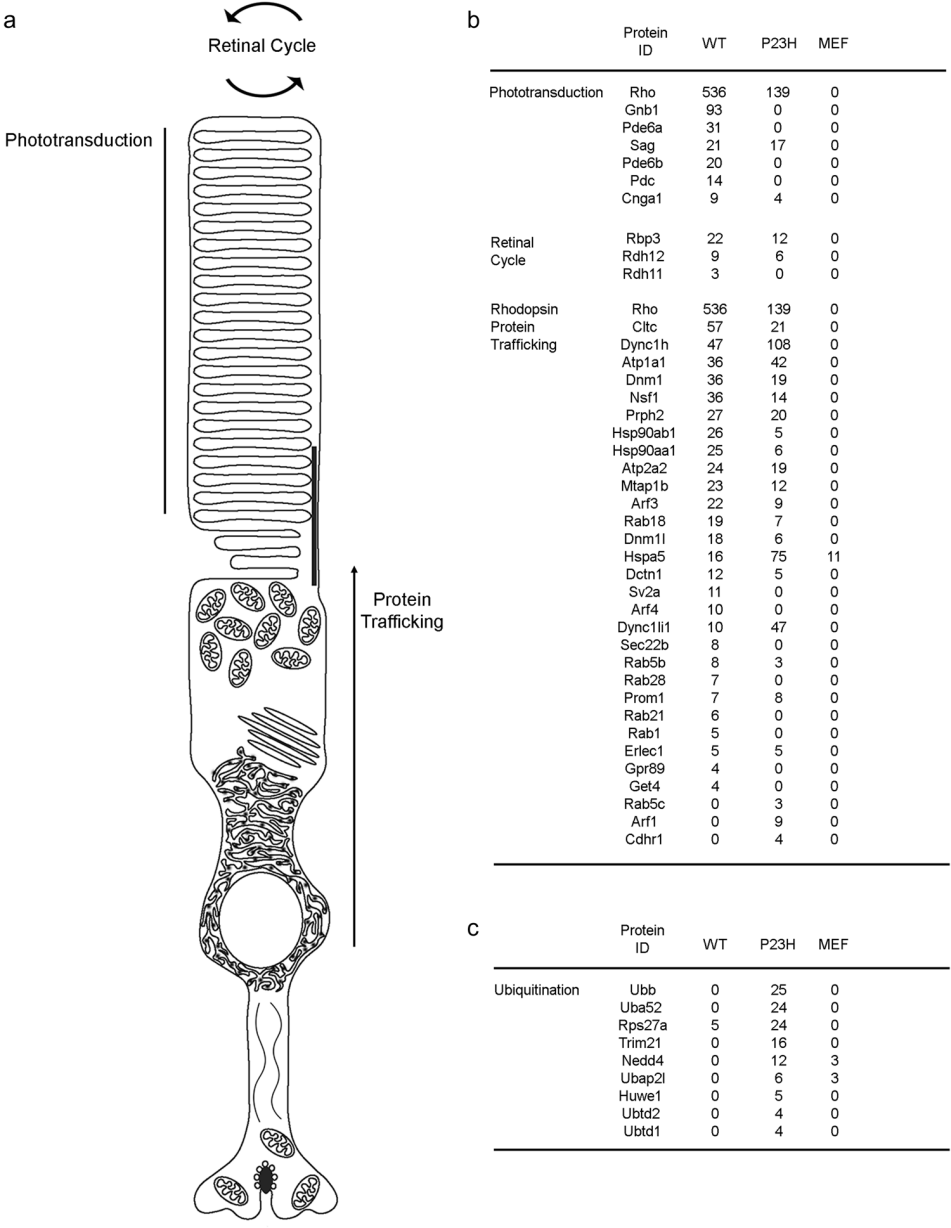
The P23H rhodopsin interactome contains decreased rod photoreceptor function proteins and increased ubiquitination proteins compared to wild-type rhodopsin interactome. (**a**) Graphic shows 3 functions unique to rod photoreceptor cells—phototransduction, vitamin A/retinal cycle, and rhodopsin protein trafficking (**b**) Spectral counts of individual proteins associated with phototransduction, retinal cycle, and rhodopsin protein trafficking identified in wild-type and P23H rhodopsin protein interactome. Spectral counts from mouse embryonic fibroblast (MEF) lysates anti-rhodopsin immunoprecipitates were analyzed by LC–MS/MS samples as a control. (**c**) Spectral counts of proteins associated with ubiquitination from wild-type rhodopsin interactome, P23H rhodopsin interactome, and MEF samples.

In wild-type photoreceptors, rhodopsin is densely packed in the outer segment in close proximity to proteins involved in phototransduction and the vitamin A retinal cycle^41^. In the wild-type rhodopsin interactome, we identified many phototransduction proteins including retinal transducin (GNB1); rod phosphodiesterase subunits (PDE6A, PDE6B); phosducin (PDC); S-arrestin (SAG); and a subunit of cyclic nucleotide gated ion channel (CNGA1) (Fig. 2a). Similarly, we also found several proteins involved in the vitamin A retinal cycle in the wild type interactome including retinol dehydrogenases (RDH11, RDH12) and retinol binding protein 3 (Rbp3) (Fig. 2a,b). By contrast, in the P23H rhodopsin interactome, spectral counts for these phototransduction and vitamin A retinal cycle proteins were markedly reduced or absent (Fig. 2a,b). In our MEF interactome control, as expected, phototransduction and retinal cycle proteins were absent (Fig. 2b). These findings support that P23H rhodopsin protein does not efficiently engage in phototransduction and vitamin A cycle and are compatible with the scotopic function defects found in Rho P23H knock-in mice^14,41^.

Rhodopsin is synthesized in the ER located in the myoid portion of the rod inner segment, and once correctly folded, rhodopsin is exported from the ER through the ellipsoid portion of the inner segment to the base of the cilia where rhodopsin is embedded into discs and delivered to the outer segment^4^. Many proteins regulate the polarized movement of rhodopsin to the outer segment including microtubule regulators, regulatory G-proteins, and vesicular trafficking^42–44^. We identified many previously described rhodopsin protein trafficking components in our wild-type rhodopsin interactome (Fig. 2a,b); by contrast, most of these trafficking components were depleted or absent for P23H rhodopsin (Fig. 2a,b). In our MEF interactome control, as expected, no rhodopsin trafficking proteins were found (Fig. 2b). Together, these findings suggested that P23H rhodopsin was unable to traffic properly to the outer segment. These studies are consistent with in vitro studies that show marked reduction of P23H rhodopsin from cell membrane surface and retention in the ER when expressed in HEK293 cells^20^; and prior *Rho*^*P23H/P23H*^ mouse studies that show little rhodopsin in the outer segment^19,45^.

Although the P23H rhodopsin interactome was markedly depleted in proteins involved in phototransduction, retinal cycle, and rhodopsin trafficking to the outer segment, we observed significant enrichment in many ubiquitination proteins including polyubiquitin precursor (UBB)^46,47^; several ubiquitin ligases (TRIM21, NEDD4, HUWE1)^48^; and ubiquitin-ribosomal fusion proteins (UBA52, RPS27A)^46,47,49^ (Fig. 2c). By contrast, none of these ubiquitination proteins, apart from RPS27A, were detected in the wild-type rhodopsin and MEF interactomes (Fig. 2c). Enrichment of ubiquitin in the P23H rhodopsin interactome is consistent with prior studies that found significant ubiquitination of P23H rhodopsin compared to wild-type rhodopsin^19^.

### Functional pathway analysis identifies translational and post-translational protein quality control mechanisms in the P23H rhodopsin interactome

Post-translational protein degradation mechanisms, ERAD and autophagy, have been implicated in P23H rhodopsin protein turnover in the retina^19,36^. To determine if these protein quality control mechanisms were present in the P23H rhodopsin protein interactome, we performed bioinformatic pathway analysis on our P23H and wild-type rhodopsin mass spectrometry datasets. We used gProfiler (https://biit.cs.ut.ee/gprofiler/)^50^ to analyze our wild-type and P23H rhodopsin protein lists across four different gene/protein function annotation databases (Supplementary Information S4 and S5): Gene Ontology Biological Processes (GO-BP)^51^; Reactome^52^; GO Molecular Functions (GO-MF)^51^; and Kyoto Encyclopedia for Genes and Genomes (KEGG)^53^.

**Figure 3:**
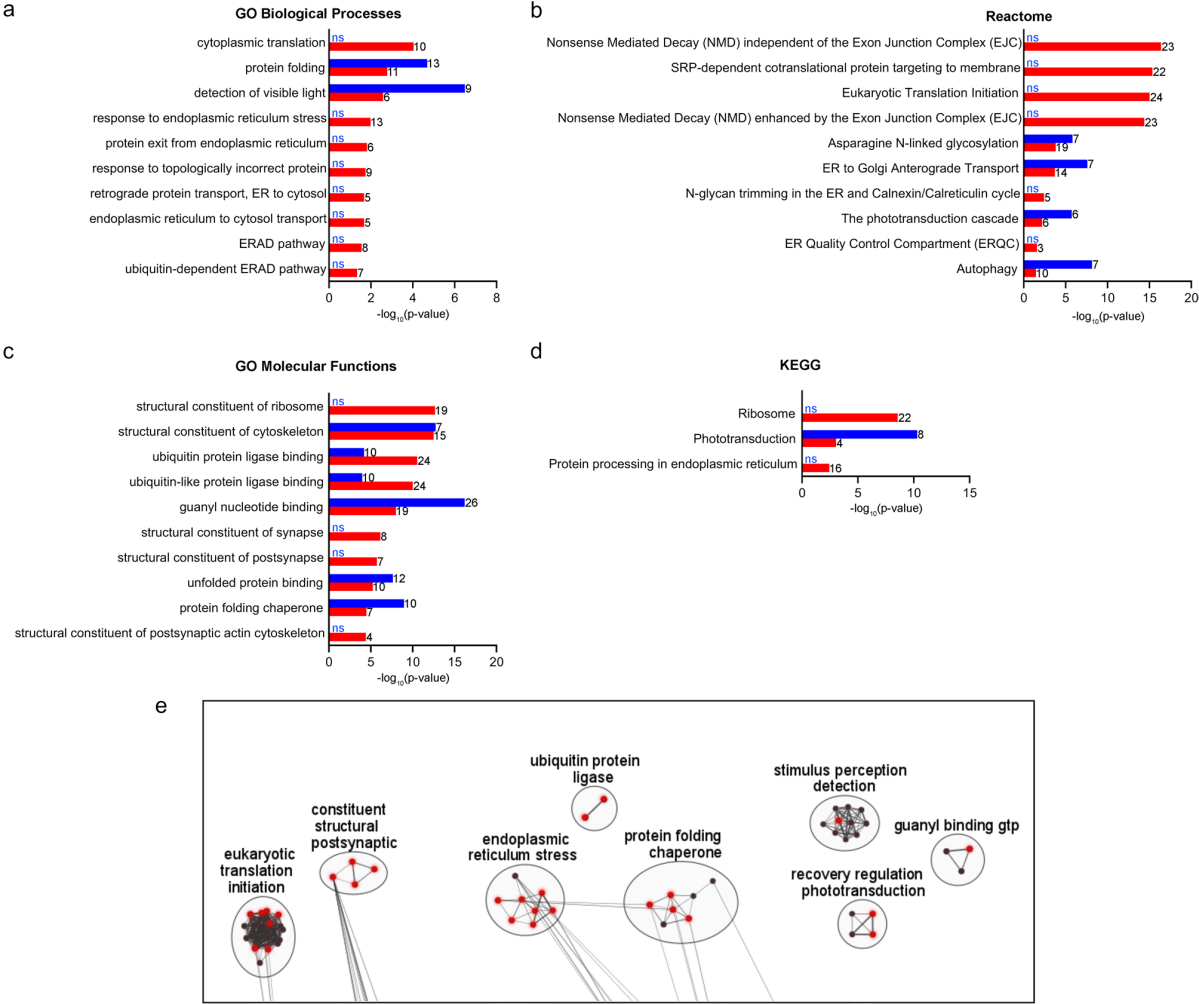
Pathway enrichment analysis using four databases shows protein quality control mechanisms significantly enriched in P23H rhodopsin interactome. (**a**) P23H rhodopsin interactome (red) shows significant enrichment in Gene Ontology (GO) biological processes terms related to translation, protein folding, endoplasmic reticulum (ER) stress, and ER-associated degradation (ERAD) not enriched in wild-type rhodopsin interactome (blue).(**b**) P23H rhodopsin interactome (red) shows significant enrichment in Reactome terms associated with mRNA decay and translation. Wild-type rhodopsin interactome (blue) shows significant enrichment in phototransduction, anterograde protein trafficking, autophagy, and glycosylation compared to P23H rhodopsin (red). (**c**) P23H rhodopsin interactome (red) shows enrichment in ribosome, ubiquitination, and synapse GO Molecular Function terms compared to wild-type rhodopsin interactome (blue). (**d**) P23H rhodopsin shows significant enrichment in ribosome and ER protein processing Kyoto Encyclopedia of Genes and Genomes (KEGG) terms not enriched in wild-type rhodopsin interactome. The number of proteins from the P23H or wild type interactome associated with each term is listed next to the bar. Ns stands for non-significant. (**e**) Common themes of the P23H rhodopsin interactome from GO biological processes, Reactome, GO molecular function, and KEGG databases are represented by clusters (black circles). Within the clusters, each dot is a node that refers to a single term from the four databases. Individual nodes are sorted into clusters and labeled by the Cytoscape plug-in, Auto Annotate. Black nodes are other terms unrepresented in a-d. Red nodes are terms presented in a-d. The complete list of nodes within these clusters are shown in Supplementary Information S7.

GO-BP analysis of the P23H rhodopsin interactome revealed significant association with translation (*p* = 9.10E-05) and many terms related to ER protein folding; ER stress; and ER-associated protein degradation (Fig. 3a). These terms were not significantly enriched in the wild-type rhodopsin interactome (Fig. 3a). By contrast, the GO-BP terms, detection of visible light and protein folding, were significantly associated with the wild-type rhodopsin interactome but had reduced association in the P23H rhodopsin interactome (Fig. 3a).

Reactome pathway analysis revealed that the P23H rhodopsin interactome was significantly associated with translation-related mechanisms including nonsense mediated decay, signal recognition particle (SRP)-dependent cotranslational protein targeting to the membrane; translation initiation; and ER quality control (Fig. 3b). By contrast, these Reactome terms were not significantly enriched in the wild-type rhodopsin interactome. Instead, the wild-type rhodopsin interactome was significantly associated with phototransduction; glycosylation; anterograde protein transport; and autophagy (Fig. 3b); all of which had reduced significance in the P23H rhodopsin interactome.

GO-MF pathway analysis of the P23H rhodopsin interactome revealed significant association with ribosome and ubiquitin protein ligase binding terms (Fig. 3c). GO-MF also identified significant enrichment in synapse-related terms in the P23H rhodopsin interactome not seen in the wild-type rhodopsin interactome (Fig. 3c). By contrast, the wild-type rhodopsin interactome showed significant association with guanyl nucleotide binding, compatible with rhodopsin’s function as a G-protein coupled receptor^1^, and protein folding chaperones (Fig. 3c); furthermore, these associations were reduced in the P23H rhodopsin interactome.

Last, KEGG pathway database analysis revealed that P23H (not wild-type) rhodopsin interacting proteins were significantly associated with ribosome and ER protein processing similar to results from GO-BP; Reactome; and GO-MF analyses (Fig. 3d). Consistent with the outputs from the other databases, P23H rhodopsin interactome showed significantly less association with phototransduction in KEGG when compared to the wild-type rhodopsin interactome (Fig. 3d).

Next, we used CytoScape (3.8.2^54^;) to identify common cellular themes among the significant terms found in the 4 databases for the P23H rhodopsin interactome (Fig. 3e). Key themes (depicted as clusters of nodes in CytoScape) unique to the P23H rhodopsin interactome included eukaryotic translation, ER stress, ubiquitination, and synapses (Fig. 3e, Supplementary Information S6, and Supplementary Information S7). Cellular themes shared with wild-type rhodopsin included photodetection/phototransduction and G-protein binding (Fig. 3e, Supplementary Information S6, and Supplementary Information S7). Put together, functional pathway analysis of P23H rhodopsin interactome revealed many processes linked to ER stress and/or ER stress-associated protein degradation, consistent with prior experimental studies of the P23H knock in mouse^19^. This analysis also identified translational regulatory events as significant, but previously unstudied, biological processes associated with P23H rhodopsin. Functional pathway analysis also reiterated P23H rhodopsin’s defect in carrying out normal photodetection and phototransduction processes when compared to wild-type rhodopsin.

**Figure 4:**
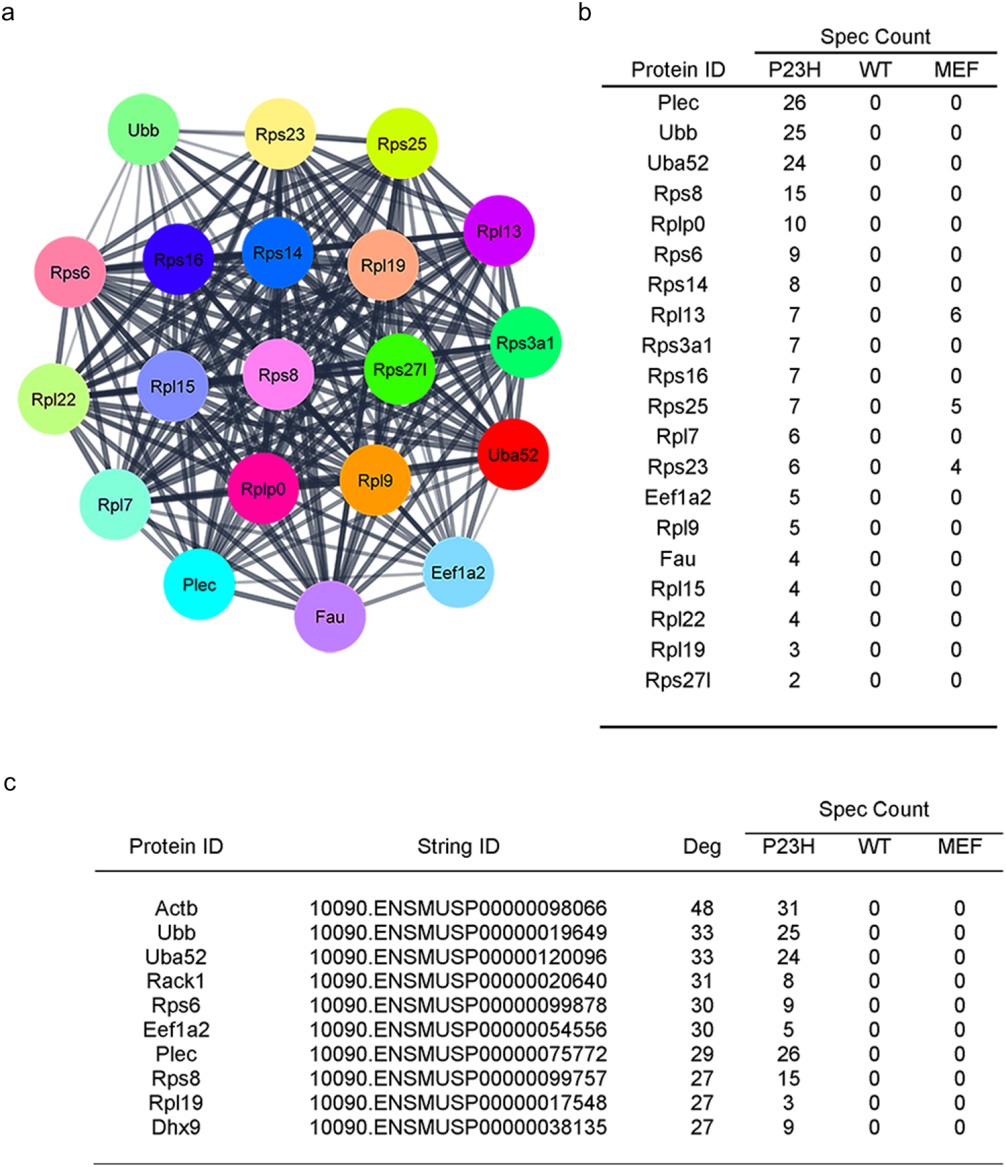
Protein–protein interaction analysis of the P23H rhodopsin interactome. (**a**) The P23H rhodopsin interactome PPI network was assembled using STRING, and MCODE was used to identify the most significant PPI module within the STRING network. The most significant PPI module (MCODE value = 21) within the P23H Rhodopsin interactome is shown. (**b**) The 20 proteins in this module and their spectral counts are listed. (**c**) Cytohubba was used to quantify the connectivity of all proteins in the P23H rhodopsin interactome STRING network, and the 10 proteins with the highest degree of connectivity and their spectral counts are listed.

To identify the important protein modules within the P23H rhodopsin interactome, we generated protein–protein interaction (PPI) networks using STRING (Search Tool for the Retrieval of Interacting Genes/Proteins) (https://string-db.org/)^55^, a database that identifies and compiles protein–protein interactions. We found 741 interactions between the 173 proteins unique to P23H rhodopsin (confidence score of > 0.400) (Supplementary Information S8. Then, PPI networks were grouped into modules using MCODE to group interconnected proteins^56^. Only one PPI module was identified at high stringency settings (MCODE > 20) composed of 20 highly interconnected proteins (Fig. 4a). Fifteen of these 20 interconnected proteins were 40S or 60S ribosome subunit components; 2 of 20, Uba52 and Fau, were fusions of ubiquitin or ubiquitin-like proteins to ribosomal proteins^49,57^; Eef1a2 was a translation elongation factor; Ubb encoded ubiquitin; and Plectin was a cytoskeletal protein. These 20 proteins were not present in the wild-type rhodopsin interactome or in the MEF mass spectrometry control experiments. These findings suggested an important and specific link between P23H rhodopsin and ribosomal functions.

To identify the top 10 most interconnected nodes (hub genes) within our network, we used cytoHubba^58^ and ranked the most interconnected proteins. Seven of the hub genes were also present in the ribosome-related PPI module identified by MCODE (Ubb, Uba52, Rps6, Eef1a2, Plec, Rps8, and Rpl19) (Fig. 4b). The remaining 3 hub genes encoded Actb (beta-actin); the RNA helicase, DHX9 (DExH-Box Helicase 9); and Rack1 (Receptor for activated C kinase 1), a ribosomal protein involved in translation, ribosome associated quality control (RQC), and mRNA decay (Fig. 4b). RQC is a protein and mRNA quality control mechanism that is activated when ribosome translation is aberrant (e.g., slowed, stalled, damaged, or collided ribosomes). Due to RQC, the ribosome-mRNA-nascent chain complex is disassembled, and mRNA promptly degraded; furthermore, the partially translated polypeptide is ubiquitinated and targeted for proteasomal degradation^59^. RQC’s role in photoreceptors and retinitis pigmentosa has not been experimentally studied. However, our identification of ribosome and RQC-regulatory proteins as top GO terms, protein interaction module, and hub genes in the P23H rhodopsin interactome raises the possibility that photoreceptors use RQC-type mechanisms to remove P23H rhodopsin protein and transcript during translation.

### Gene ontology cellular compartment analysis supports synaptic mislocalization of P23H rhodopsin

**Figure 5:**
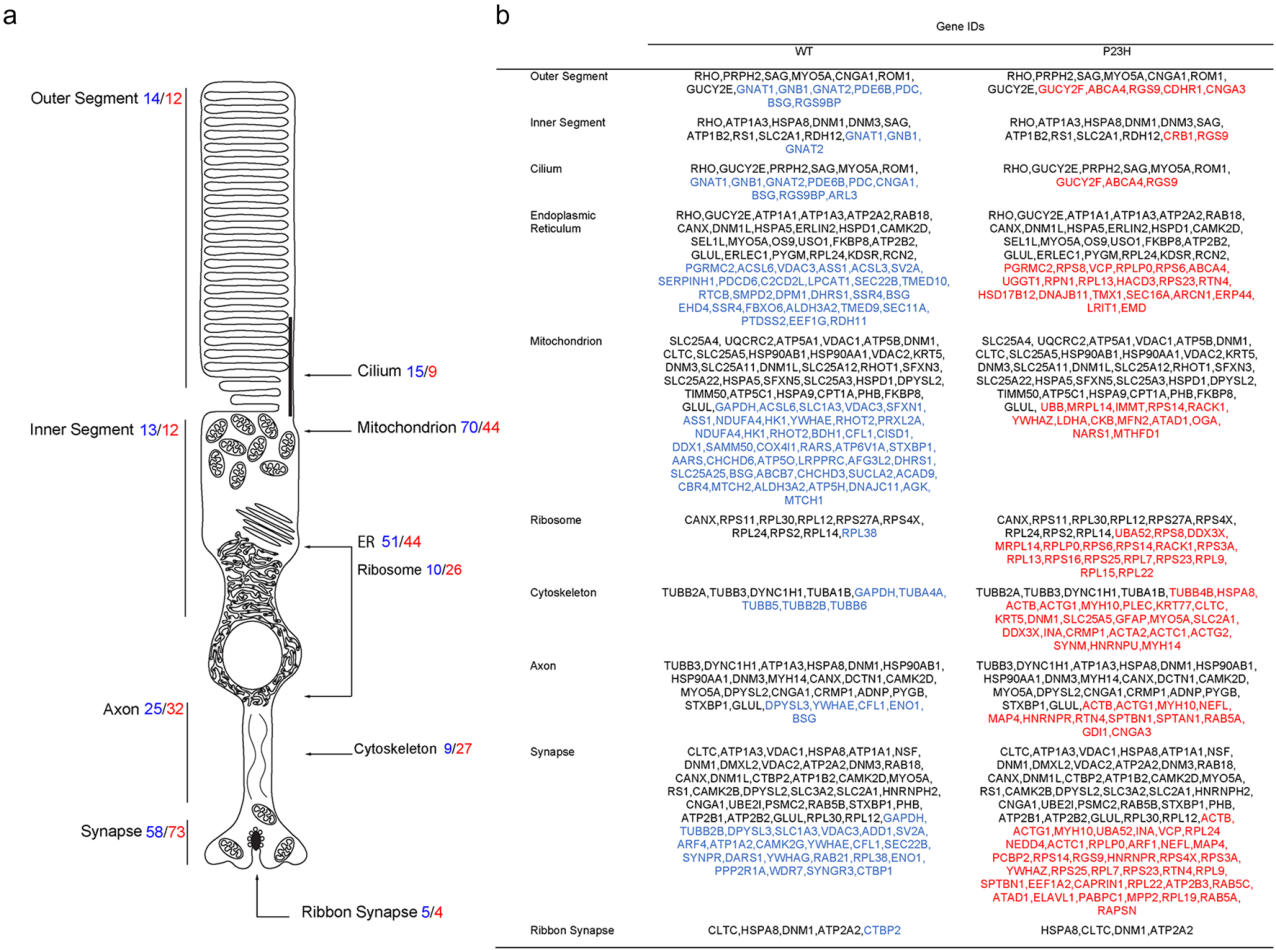
P23H rhodopsin interactome shows enrichment of proteins from synaptic, axonal, cytoskeletal, and ribosome compartments. (**a**) Graphic shows several subcellular structures in the rod photoreceptor cell. The number of proteins associated with wild-type (blue) or P23H (red) rhodopsin significantly found at each structure by Gene Ontology analysis are shown. (**b**) The table shows individual proteins associated with wild-type (blue) or P23H (red) rhodopsin significantly associated with specific subcellular structures. Common proteins (found in both wild-type and P23H rhodopsin interactome) are shown in black.

Normally, rhodopsin is efficiently transported unidirectionally from the ER to the rod outer segment (ROS)^43^. However, studies in mice carrying RFP-tagged P23H rhodopsin found that the RFP-tagged P23H rhodopsin was aggregating in the ER and mislocalized to regions such as the axons and axon terminals^16^. In our P23H rhodopsin proteomic dataset, GO-MF analysis also identified significant enrichment in synaptic terms (Fig. 3c). To further determine if P23H rhodopsin was mislocalized to axons and axon terminals in our dataset, we used the gene ontology cellular compartment (GO-CC) database to identify the subcellular distribution of P23H rhodopsin interacting proteins compared to that of wild-type rhodopsin. We found a marked increase of P23H rhodopsin interacting proteins in synapse, cytoskeleton, and axon compartments compared to wild-type rhodopsin (Fig. 5a,b). These are compatible with defective anterograde transport of P23H to the ROS and mislocalization toward the ribbon synapse. We also found marked increase in P23H rhodopsin interacting proteins in the ribosome compared to wild-type rhodopsin (Fig. 5a,b), consistent with identification of ribosomal processes as top hits in the P23H rhodopsin interactome network analyses (Figs. 3, 4).

## Discussion

**Figure 6:**
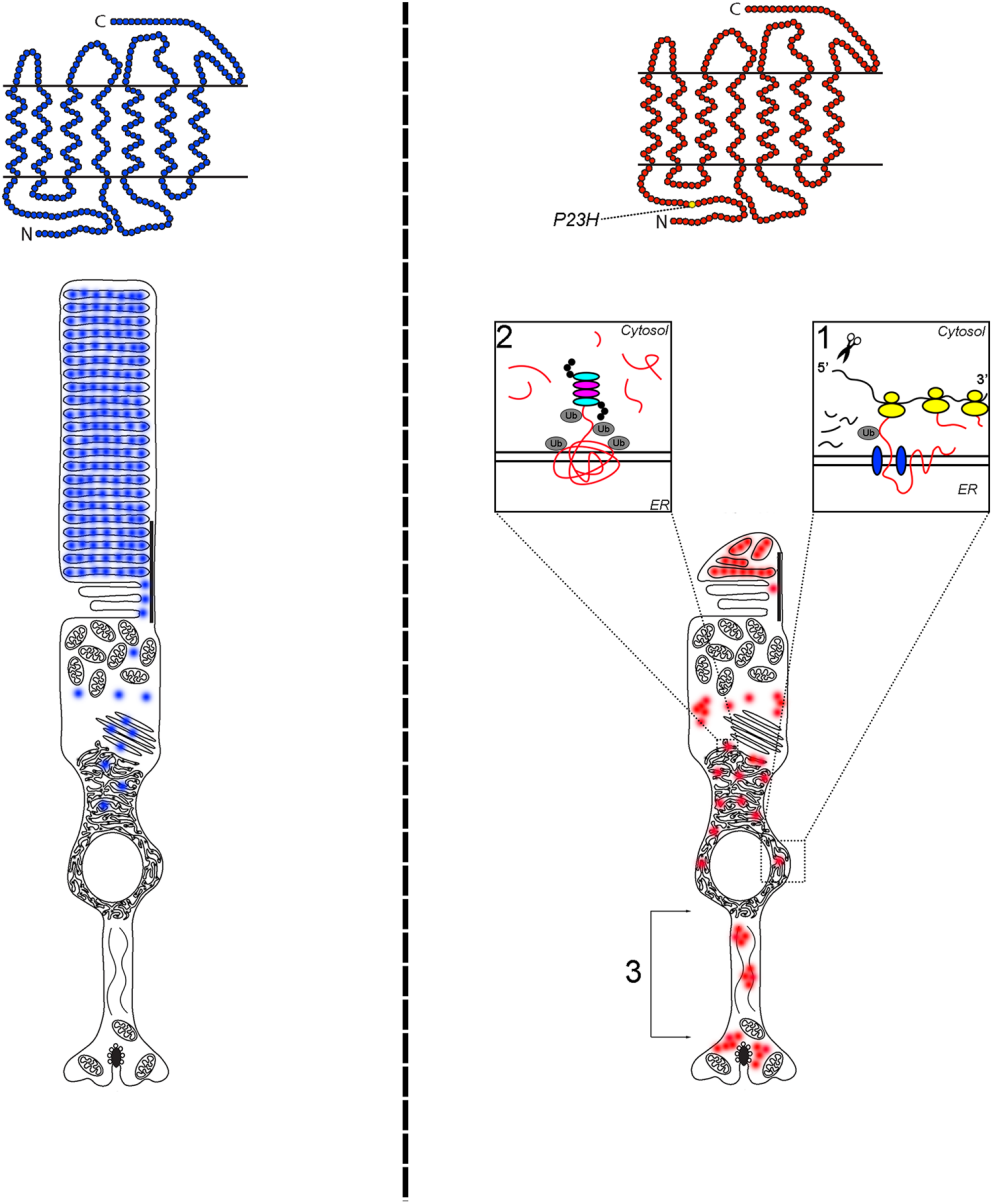
Model of protein and mRNA quality control mechanisms identified in P23H rhodopsin interactome. On the left, wild-type rhodopsin (blue) is translated, folded, and processed at the ER, and transported anterograde to the rod outer segment. On the right, (1) *P23H rhodopsin* mRNA and nascent P23H rhodopsin protein are targeted for degradation through co-translational quality control mechanisms such as ribosome-quality control and mRNA decay. (2) fully synthesized P23H rhodopsin protein (that escapes quality control steps during translation) is targeted for degradation through post-translational quality control mechanisms such as ERAD. ERAD retrotranslocates fully synthesized P23H rhodopsin from ER to cytosol for ubiquitination and proteasomal degradation. (3) Any remaining P23H rhodopsin protein that escapes translational and post-translational quality control steps progresses to outer segment and abnormally accumulates in synapse.

Many heritable retinal degenerative diseases are caused by rhodopsin variants that introduce missense changes and, ultimately, protein misfolding^17^. Rods rapidly eliminate misfolded rhodopsin proteins produced by rhodopsin disease alleles, but the catabolic mechanisms by which rods identify and remove mutant rhodopsin are not well understood. Here, we used mass spectrometry to identify and compare protein interaction partners of P23H rhodopsin and wild-type rhodopsin purified from mouse retina. We found substantial differences in protein-interacting partners between P23H and wild-type rhodopsin (Fig. 1e, Supplementary Information S2, Supplementary Information S3). As expected, proteins involved in normal rod functions including phototransduction, retinal cycle, and rhodopsin trafficking to outer segment were significantly reduced in P23H rhodopsin interactome compared to that of wild-type rhodopsin. By contrast, the P23H rhodopsin protein interactome was enriched in ubiquitination, protein degradation, and synapse components compared to wild-type rhodopsin. GO analysis of the P23H rhodopsin interactome identified the post-translational protein quality control mechanism, ER stress-associated protein degradation, as a significant process, consistent with prior studies demonstrating the induction of ER stress and UPR by misfolded rhodopsin^60^. Interestingly, GO and network analysis also identified additional quality control steps that operate during translation (ribosome quality control and mRNA decay) as significantly enriched processes in P23H rhodopsin protein interactome. A model to reconcile the multiple different quality control processes identified in the P23H rhodopsin interactome is that P23H rhodopsin mRNA and nascent partially synthesized protein are targeted for degradation by translational quality control mechanisms (Fig. 6). P23H rhodopsin mRNA and protein that escapes translational quality control mechanisms to produce full-length P23H rhodopsin protein in the ER is subsequently targeted by post-translational quality control mechanisms, such as ERAD, to remove the mutant protein from photoreceptors (Fig. 6). Since photoreceptors transcribe and translate massive amounts of rhodopsin, multiple quality control tiers may be necessary to fully remove mutant dysfunctional rhodopsin.

Our study provides the first proteomic analysis of wild-type and mutant rhodopsin protein interaction partners isolated from native retina, and these datasets provide a resource for further investigation of rhodopsin in rod health and retinal degeneration. Several limitations should be kept in mind in interpreting our wild-type and P23H rhodopsin protein interactome datasets. First, our current datasets do not capture the complete wild-type and P23H rhodopsin proteins (Table 1). We recovered ~ 1/3 of the rhodopsin protein sequence by mass spectrometry (Table 1); the remaining ~ 2/3 of rhodopsin was not detected in our study. Rhodopsin is a 7 transmembrane G-protein-coupled receptor, and the targets for tryptic cleavage, lysine and arginine, are rare in transmembrane helices, which would limit the ability to identify full sequence coverage for rhodopsin. Additional methods for enzymatic digestion of membrane proteins can be tested on rhodopsin in future studies. Second, our rhodopsin interactome datasets were based on analysis of P15 mouse retinas. Rhodopsin-interacting proteins expressed at other ages would be missed in our study. A third limitation of our study is that we may be identifying non-physiologic rhodopsin protein interaction partners because we used whole retina protein lysates in our rhodopsin immunoprecipitations. Spatial proteomic retinal profiling experiments may be useful next steps to evaluate if the interacting proteins identified in our mass spectrometry studies are found in rod cells (where rhodopsin is selectively expressed) or RPE (where ROS carrying rhodopsin are phagocytosed). Although the sensitivity of our approach to identify rhodopsin interacting proteins carries the aforementioned caveats, we believe the specificity of the wild-type or P23H rhodopsin-interacting proteins identified in our study is high, because few, if any, of these proteins appeared in control mass spectrometry experiments performed on immunoprecipitates from MEF lysates. However, MEF and retina protein lysates fundamentally differ. Additional controls to determine immunoprecipitation specificity in future studies could include use of protein lysates from rhodopsin knockout mice, beads without antibody, or control non-specific antibodies.

Post-translational quality control mechanisms, ERAD and autophagy, clear P23H rhodopsin in cell culture models^19,20,28^; and are also active in the retina^17,19,36^. In our P23H rhodopsin interactome dataset, we found support that ER stress and ERAD mechanisms are significantly associated with Rho^P23H/P23H^ through significant enrichment of ERAD, ER stress, and other ER-related terms. In addition, individual proteins experimentally validated as important mediators of ER stress and ERAD like BiP/Grp78 and VCP were either more prominent in the P23H rhodopsin interactome or only found associated to P23H rhodopsin^23,61^. By contrast to ERAD, we did not find significant support for autophagy in our P23H rhodopsin interactome ^36^. We speculate that the induction of ERAD, as found in our data, may lessen the need for autophagy in P23H rhodopsin turnover^62^, and this could explain the absence or lack of enrichment in autophagy terms in the P23H rhodopsin interactome. While we saw no enrichment of autophagy in the P23H rhodopsin interactome, we did observe enrichment of autophagy in the WT rhodopsin interactome (Fig. 3). In healthy photoreceptors, RPE cells phagocytose and degrade the ROS through the lysosome^4^. Autophagy also degrades proteins through the lysosome. The enrichment of autophagy terms in the wild-type rhodopsin interactome may reflect the use of lysosomal components involved in ROS degradation by RPE^63^. By contrast, RPE-mediated phagocytic degradation of ROS and delivery of P23H rhodopsin to the lysosome is less significant in *Rho*^*P23H/P23H*^ mice since their ROS are severely stunted and contain little rhodopsin (Fig. 1)^19,45^.

Surprisingly, our P23H rhodopsin protein interactome contained numerous proteins involved in ribosomal translation. Although ribosomal proteins are abundant, few of these proteins were found in the WT rhodopsin interactome and MEF control experiments. This suggested that P23H rhodopsin selectively engages with ribosomal functions. Pathway enrichment and network analysis implicated specific ribosome translational quality control mechanisms and regulators, RACK1 and UBA52, in the P23H rhodopsin interactome. RACK1 is an essential component of the 40S ribosomal subunit and has multiple functions in translational quality control. RACK1 can recognize incorrect mRNA sequences during translation and initiate the cleavage of the aberrant mRNA^59,64^. RACK1 also plays a significant role towards ubiquitination of the ribosome and initiation of RQC to dissemble translating ribosomes and degrade nascent partially-translated polypeptides and aberrant mRNA^64,65^. UBA52 is a fusion protein with ubiquitin at the C-terminus and ribosomal protein L40 at the N-terminus^66^. UBA52 provides ubiquitin to the ribosome and regulates ribosomal ubiquitination^67^. Interestingly, recent studies indicate that ER stress causes ubiquitination of the ribosome and activation of RQC and UPR genes like IRE1 are associated to the RQC inducing event, ribosome collision^68,69^. The significance of RQC proteins like RACK1 and UBA52 have not been experimentally examined in photoreceptors. However, RQC could be a useful mechanism to remove P23H rhodopsin from rods because RQC targets both nascent proteins and encoding mRNAs for degradation when the ribosomal complex is dissembled. With respect to P23H rhodopsin, RQC could contribute to the elimination of P23H rhodopsin during translation and target *P23H rhodopsin* mRNA for nucleolytic cleavage to prevent synthesis of further misfolded protein. Interestingly, previous studies found that mRNA levels of P23H rhodopsin are lower than WT rhodopsin in the same mice although the copy numbers of the two mice are not significantly different^14,70^.

Aside from translational and post-translational quality control mechanisms, we also found significant and selective association of P23H rhodopsin interactome with synaptic processes. This supports prior studies that found mislocalization of P23H rhodopsin at the synapse^16,71^. Mislocalized P23H rhodopsin may reflect protein that escapes degradation at the ER or during translation.

Modulation of protein quality control has shown therapeutic promise in preventing retinal degeneration arising from rhodopsin mutations. Here, we provide proteomic evidence that ERAD is an important protein quality control mechanism activated by photoreceptors expressing P23H rhodopsin protein. Our data also suggests that photoreceptors employ additional quality control mechanisms, besides ERAD, that operate earlier during ribosomal translation of P23H rhodopsin. Further studies can elucidate the significance and mechanism of translational quality control in P23H rhodopsin elimination and in influencing rod photoreceptor cell viability and disease.

## Experimental procedures

### Animals

*Rho*^*P23H/P23H*^ or *Rho*^+*/*+^ mice in C57BL/J6 background were used for this study. *rd8* absence was confirmed in mice by RT-PCR^72^. Mice were euthanized at P15 through carbon dioxide euthanasia followed by cervical dislocation, and eyes were enucleated from *Rho*^+*/*+^ and *Rho*^*P23H/P23H*^ mice and processed for histology or biochemistry (immunoblotting and immunoprecipitation). All mouse care and experimental procedures in this study were approved and conducted in strict accordance with relevant guidelines and regulations by the Institutional Animal Care and Use Committee at Stanford University and in compliance with the Association for Research in Vision and Ophthalmology Statement for the Use of Animals in Ophthalmic and Vision Research and the ARRIVE (Animal Research: Reporting of in Vivo Experiments) guidelines.

### Histology

Enucleated eyes were processed for histology by immersing them in a fixative of mixed aldehydes^19,73^. After fixation, eyes were bisected, post-fixed in osmium tetroxide, and embedded in epoxy resin^19,73^. One-micrometer-thick sections were cut through the optic nerve head along the vertical meridian and stained with toluidine blue^19,73^. Slides were photographed by a light microscope (20 × objective, Zeiss Imager Z1 light microscope, Thornwood, NY). Light micrographs were collected to analyze and compare the outer segment, inner segment, and outer nuclear layer from *Rho*^+*/*+^ and *Rho*^*P23H/P23H*^ mice retinas.

### Immunoblotting

Retinas were dissected from enucleations and lysed with 300 µl lysis buffer containing maltoside (PBS, 0.5 g/ml n-Dodecyl-β-D-maltoside (Calbiochem EMD Bioscience, San Diego, CA), protease inhibitors (Sigma-Aldrich, St. Louis, MO), and phosphatase inhibitor (Thermo Scientific, Rockford, IL). 1D4 anti-rhodopsin antibody (Santa Cruz Biotechnologies, Santa Cruz, CA) at 1:1000 dilution was used to perform the western blot. Membranes were washed and incubated overnight with 1D4 antibody. The following day, membranes were incubated with a horseradish peroxidase-coupled secondary antibody (Cell Signaling, Danvers, MA). Immunoreactivity was detected with SuperSignal West chemiluminescence substrate (Pierce ThermoFisher, Waltham, MA).

### Immunoprecipitation

Rhodopsin proteins were immunoprecipitated to perform mass spectrometry analysis. *Rho*^+*/*+^ and *Rho*^*P23H/P23H*^ retinas were dissected from enucleations and cultured MEFs were scraped and pelleted. Retinas and MEF cells were lysed in 1 ml of PBSD (PBS containing 1.0% n-dodecyl-β-D-maltoside and protease and phosphatase inhibitor mixture) and sonicated. Supernatants were collected and protein concentrations were determined by BCA (Pierce Thermo Fisher, Waltham, MA). Equal levels of proteins were then added to the Dynabeads Protein G (Invitrogen Thermo Fisher, Waltham, MA); preincubated with 1D4 anti-rhodopsin antibody (Santa Cruz Biotechnologies); and incubated at 4 °C overnight. Protein eluates for immunoblotting were collected from the beads heated with SDS sample buffer at 80 °C. Protein eluates for mass spectrometry were eluted from the beads using an elution buffer (PBS containing 8 M urea and 0.5% n-dodecyl-β-D-maltoside) at 80 °C, and detergent was removed by methanol chloroform extraction.

### Liquid chromatography tandem mass spectrometry

Protein eluates from the *Rho*^+*/*+^, *Rho*^*P23H/P23H*^, and MEF immunoprecipitates were resuspended in 8 M urea. The samples were reduced by incubation with 5 mM tris(2-carboxyethyl) phosphine for 20 min at room temperature and alkylated in the dark by treatment with 10 mM iodoacetamide for 15 min. Proteins were digested overnight at 37 °C with Sequencing Grade Modified Trypsin (Promega, Madison, WI). Proteolysis was stopped by acidification. A 100- µm i.d. capillary with a 5-µ m pulled tip was packed with 10 cm of 5-µm Aqua C18 material (Phenomenex). Then a desalting column was used to equilibrate for 30 min with 5% Acetonitrile/0.1% formic acid. The protein digest was loaded under pressure into the column in line with an Agilent 1200 quaternary HPLC and analyzed after elution and separation. As peptides eluted from the microcapillary HPLC column, they were electro-sprayed directly onto an LTQ-Orbitrap mass spectrometer from Thermo Finnigan (Waltham, MA) with the application of a distal 2.4-kV spray voltage. Protein identification, quantification, and analyses were done with Integrated Proteomics Pipeline (IP2, Integrated Proteomics Applications, Inc., www.integratedproteomics. com/) using ProLuCID and DTASelect2. Spectrum raw files were extracted into ms1 and ms2 files from raw files with Raw Extract 1.9.9 (http://fields.scripps.edu/downloads.php), and the tandem mass spectra were searched against European Bioinformatic Institute protein databases. Tandem mass spectra were matched to sequences by using the ProLuCID algorithm with 50 ppm peptide mass tolerance for precursor ions and 400 ppm for fragment ions.

### Pathway enrichment analysis

gProfiler, a web-based application (https://biit.cs.ut.ee/gprofiler/), was used for enrichment analysis. gProfiler assesses functional analysis of gene lists that integrate various sources of information which includes Gene Ontology (GO) [Biological Processes (BP), Molecular Function (MF), and Cellular Component (CC)], KEGG pathways, and Reactome. The protein identification results from the *Rho*^*P23H/P23H*^ and *Rho*^+*/*+^ mass spectrometry experiments were used as the input sets, and terms with significant (*p* <  0.05) association were collected. The gene enrichment map file, which includes significant terms from all databases, produced by gProfiler was imported into Cytoscape and visualization of all terms into enrichment map was done by Cytoscape plug-in, Enrichment Map^74^. Terms were grouped together into clusters by Cytoscape plug-in, clusterMaker2; creation of clusters is dependent on similarity of node attributes (I.e. overlapping genes)^75^. The clusters created were labeled by Cytoscape plug-in, Auto Annotate; labeling is dependent on word frequencies of the terms^75^.

### Protein–protein interaction network construction and hub gene identification

The Search Tool for the Retrieval of Interacting Genes (STRING) biological database^55^ was used to construct a protein–protein interaction (PPI) network based on the genes within the P23H rhodopsin interactome. Using STRING, PPIs were constructed with a confidence score ≥ 0.4 (default value). Subsequently, the PPI network was visualized by means of Cytoscape software (version 3.8.2). Furthermore, the plug-in of Molecular Complex Detection (MCODE)^56^ in Cytoscape software was applied to identify significant modules in the STRING PPI network. MCODE parameters were set as degree cutoff = 2, K-Core = 2, and Node Score Cutoff = 0.2. Finally, discovery of hub genes was determined by plug-in of cytoHubba in Cytoscape software; the network scoring method, the degree method, was used to determine which 10 proteins had the highest connectivity^58^.

## Supplementary Information

Supplementary Information 1.

Supplementary Information 2.

Supplementary Information 3.

Supplementary Information 4.

Supplementary Information 5.

Supplementary Information 6.

Supplementary Information 7.

Supplementary Information 8.

## Data Availability

The raw MS files have been deposited in the ProteomeXchange Consortium (http://proteomecentral.proteomexchange.org/cgi/GetDataset) through the MassIVE (https://massive.ucsd.edu/ProteoSAFe/static/massive.jsp) partner repository with the dataset identifier MSV000090197 (ftp://massive.ucsd.edu/MSV000090197/).
