# Supplementary figures and images for: Network biology analysis of P23H rhodopsin interactome identifies protein and mRNA quality control mechanisms
Source: Sci Rep. 2022 Oct 18;12:17405. doi: 10.1038/s41598-022-22316-8 (PMC9579138; doi:10.1038/s41598-022-22316-8)

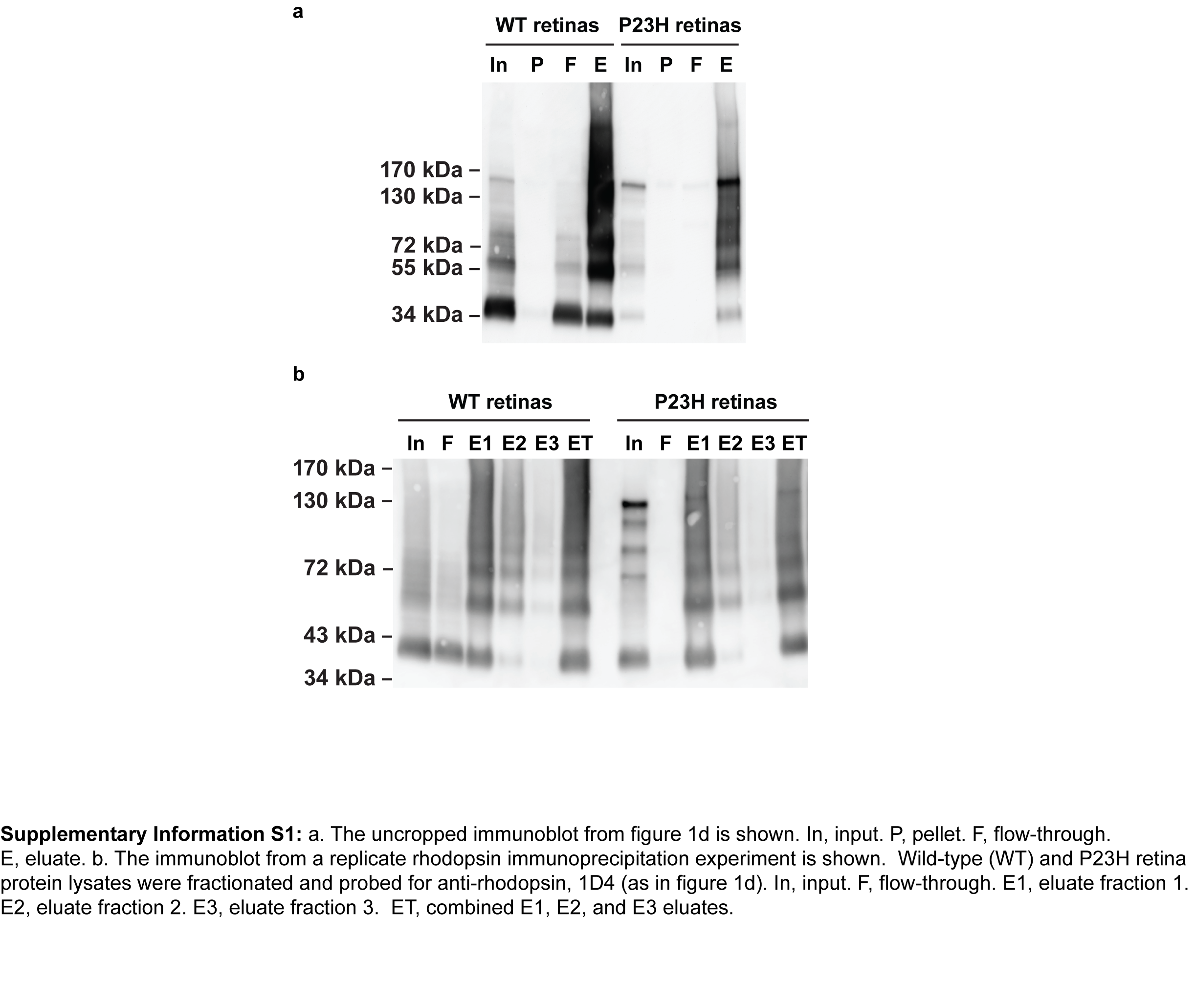

Supplement: Supplementary file 1 — Supplementary Information 1. [file 41598_2022_22316_MOESM1_ESM.tif]

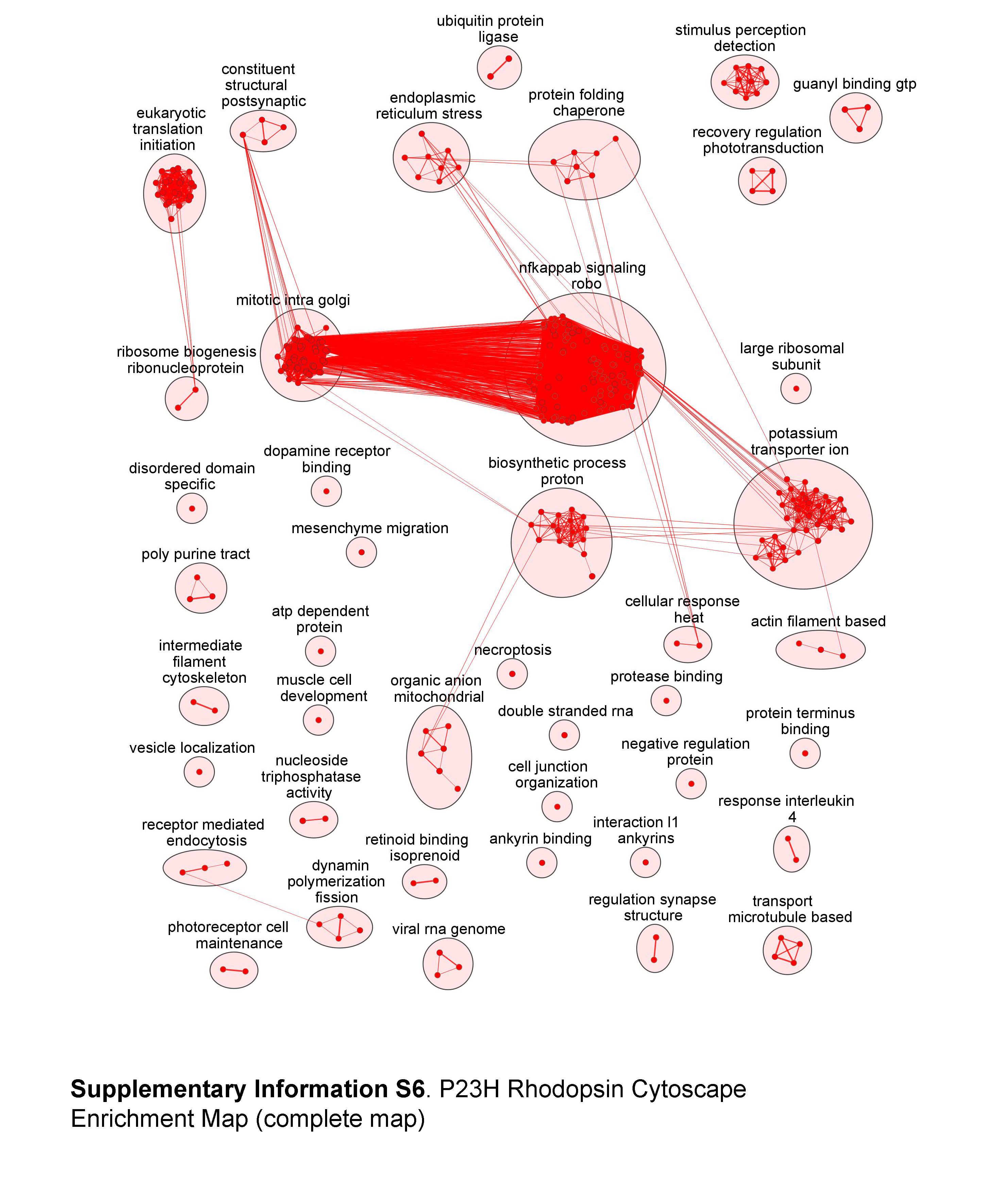

Supplement: Supplementary file 6 — Supplementary Information 6. [file 41598_2022_22316_MOESM6_ESM.tif]
